# Thorium for Pyelography

**Published:** 1916-10

**Authors:** 


**Thorium for Pyelography.** J. Edward Burns, Bull. Johns Hopkins Hosp., June, states that thorium nitrate, up to 50% in solution, is an ideal injection medium for pathologic and anatomic specimens intended for X-ray examination, the metal being next to mercury in atomic weight and the solutions being especially adapted to the demonstration of fine vessels. He illustrates with a cut of the rena pelvis and ureter and of the seminal vesicles, ampullae and vasa deferentia of rabbits, prepared with injection of 15% thorium nitrate and fixation with Kaiserling's solution which precipitates thorium in the walls of the ducts. The objection to thorium nitrate is its irritant toxicity. For clinical use, he dissolves thorium nitrate in as little distilled water as possible, keeps it hot on a water or steam bath, adds 30 c.c. of 50% solution of sodium citrate, a little at a time, with agitation. A white, gummy precipitate forms, becoming granular and finally dissolving when all of the sodium citrate has been added. Normal solution of sodium hydroxid is added to make the solution neutral to litmus and the whole made up to 100 c.c with distilled water. This is filtered and can be sterilized by heat.

